# E3 ligase TRIM15 facilitates non-small cell lung cancer progression through mediating Keap1-Nrf2 signaling pathway

**DOI:** 10.1186/s12964-022-00875-7

**Published:** 2022-05-09

**Authors:** Manman Liang, Lijing Wang, Zhengui Sun, Xingwu Chen, Hanli Wang, Lilong Qin, Wenying Zhao, Biao Geng

**Affiliations:** 1 Department of Internal Medicine, Yijishan Hospital, The First Affiliated Hospital of Wannan Medical College, Wuhu, 241000 Anhui China; 2 Department of Respiratory Medicine, Yijishan Hospital, The First Affiliated Hospital of Wannan Medical College, 2 Zheshan West Road, Wuhu, 241000 Anhui China; 3 Department of Medical Oncology, Yijishan Hospital, The First Affiliated Hospital of Wannan Medical College, Wuhu, 241000 Anhui China

**Keywords:** NSCLC, TRIM15, Keap1, Nrf2, Ubiquitination and degradation

## Abstract

**Background:**

Recent studies have indicated that some members of the tripartite motif (TRIM) proteins function as important regulators for non-small cell lung cancer (NSCLC), However, the regulatory mechanism underpinning aberrant expression of TRIM in NSCLC remains unclear. Here we report that TRIM15 plays important roles in NSCLC progression through modulating Keap1-Nrf2 signaling pathway.

**Methods:**

TRIM15 expression was evaluated by western blot analysis, tissue microarray-based immunohistochemistry analysis. The interactions between TRIM15 and Keap1 were analyzed by co-immunoprecipitation (Co-IP) and immunofluorescence co-localization assay. The correlation between TRIM15 and Keap1 was measured by Co-IP and ubiquitination analysis in vitro. Gain- and lost-of-function experiments were used to detect TRIM15 promotes proliferation and invasion of NSCLC cells both in vitro and vivo.

**Results:**

Here, we revealed that TRIM15 was frequently upregulated in NSCLC samples and associated with poor prognosis. Functionally, TRIM15 knockdown resulted in decreased cancer cell proliferation and metastasis, whereas ectopic TRIM15 expression facilitated tumor cancer cell proliferation and metastasis in vitro and in vivo. Moreover, TRIM15 promoted cell proliferation and metastasis depends on its E3 ubiquitin ligase. Mechanistically, TRIM15 directly targeted Keap1 by ubiquitination and degradation, the principal regulator of Nrf2 degradation, leading to Nrf2 escaping from Keap1-mediated degradation, subsequently promoting antioxidant response and tumor progression.

**Conclusions:**

Therefore, our study characterizes the pivotal roles of TRIM15 promotes NSCLC progression via Nrf2 stability mediated by promoting Keap1 ubiquitination and degradation and could be a valuable prognostic biomarker and a potential therapeutic target in NSCLC.

**Video Abstract**

**Supplementary Information:**

The online version contains supplementary material available at 10.1186/s12964-022-00875-7.

## Introduction

Lung cancer remains the leading cause of cancer-related death worldwide. Although the recent outstanding improvements in the survival rates due to tyrosine kinase inhibitors and immunotherapy, only 16% of patients affected by non-small cell lung cancer survive the disease after 5 years from diagnosis, with most patients being diagnosed at advanced stages characterized by metastatic dissemination [1–3].

Nuclear factor, erythroid 2-like transcription factor 2 (Nrf2) and its cytoplasmic inhibitor, kelch-like ECH-associated protein 1 (Keap1), comprise a redox-responsive endogenous antioxidant defense module that orchestrates the expression of cytoprotective genes to maintain homeostasis [4, 5]. The Keap1-Nrf2 pathway plays a protective role in many diseases where oxidative stress is thought to play an essential role in disease onset and progression, including cancer, aging-related diseases, and inflammatory diseases [6, 7]. While Nrf2 and its target genes provide protection against various age-related diseases including tumorigenesis, constitutively active Nrf2 in cancer cells increases the expression of cytoprotective genes and, consequently, enhances proliferation via metabolic reprogramming and inhibition of apoptosis [7–9]. It is widely accepted that the Keap1-Nrf2 signaling pathway is associated with the proliferation of cancer cells and tumorigenesis through metabolic reprogramming [8]. Understanding more integrated Keap1-Nrf2 mediated tumor progression may facilitate the discovery of new anti-cancer treatment strategies.

Emerging clinical evidence shows that the deregulation of ubiquitin-mediated degradation of oncogene products or tumor suppressors is likely to be involved in the aetiology of carcinomas. Recent studies have indicated that some members of the TRIM proteins (one of the subfamilies of the RING type E3 ubiquitin ligases) function as important regulators for carcinogenesis [10–12]. The TRIM family proteins, contain three highly conserved domains, which consist of a common N-terminal Really Interesting New Gene finger domain, one or two B-box motifs and a coiled-coil region [13]. TRIM family proteins are involved in a broad range of biological processes and their alterations are associated with diverse pathological conditions, such as transcriptional regulation, cell growth, apoptosis, development and tumorigenesis [13, 14]. Most of the TRIM proteins function as E3 ubiquitin ligases, and several TRIM family members are involved in various oncogenic processes, such as transcriptional regulation, cell proliferation and apoptosis. For example, TRIM32 promotes squamous cell carcinoma progression by degrading ARID1A [15]. Altered TRIM25 activity causes coordinated broad changes in the metastatic program, affecting the expression of many metastasis effectors simultaneously and promoting the metastatic phenotype [16]. In addition, previous studies from our laboratory demonstrated that TRIM59 converts macrophages to tumor-promoting functions of macrophages via regulating ABHD5 proteasomal degradation, to activate NLRP3 inflammasome signaling pathway to promote lung cancer progression by IL-1β secretion [17]. Accumulating evidence suggests that TRIM15 has a key role in many physiological disorders, predominantly by regulating ubiquitination of its target protein [18, 19]. For example, TRIM15 is required for the growth of both drug-responsive and drug-resistant melanoma cells [18]. Mechanistically, TRIM15 is crucially involved in ERK pathway by mediating K63-linked polyubiquitination of ERK1/2, which is important for ERK interaction with and activation by MEK. However, the molecular mechanism underlying TRIM15-mediated malignancies the signaling function of TRIM15 have not been elucidated.

In this study, we reported that TRIM15 was significantly upregulated in NSCLC and that increased TRIM15 was associated with poor survival. Gain- and loss-of-function experiments showed that TRIM15 promoted tumor proliferation and metastasis by activating Nrf2 signaling. Mechanistically, TRIM15 destabilized Keap1 via enhancing its ubiquitination and degradation, and accordingly prevented degradation of Nrf2 resulting in activation of Nrf2 pathway, enhanced tumor proliferation and metastasis. This study provides insights into TRIM15-dependent regulation of tumorigenesis, progression, and metastasis in NSCLC.

## Materials and methods

### Cell lines and cell culture

The human lung cancer cell lines A549, H1299, HCC827, H1975, H460, H1650 and embryonic kidney (HEK293) cell lines were purchased from the Cell Bank of Type Culture Collection of the Chinese Academy of Sciences Shanghai Institute of Biochemistry and Cell Biology. Cells were cultured in high-glucose DMEM (DMEM-HG) containing 10% FBS and were incubated at 37 °C in a humidified atmosphere with 5% CO_2_. All cell lines were authenticated by the DNA finger printing analysis and tested to be free of mycoplasma infection. These cell lines were Mycoplasma-free and routinely authenticated by quality examinations of morphology and the growth profile.

### Western blot analysis

Total proteins extracted from the cells was separated by 8–12% SDS-PAGE and transferred onto nitrocellulose membranes, which were incubated with specific antibodies overnight at 4 °C, probed with HRP-conjugated secondary antibodies, and visualized using enhanced chemiluminescence reagent (Pierce). β-actin was used as the loading control. The following antibodies were used: TRIM15 (Cat: PA5-40946, Invitrogen), Keap1 (Cat: PA5-99434, Invitrogen), Nrf2 (Cat: PA5-27882, Invitrogen), anti-6x-His (Cat: MA1-21315, Invitrogen), anti-HA (Cat: 32-6700, Invitrogen), NQO1 (Cat: MA1-16672, Invitrogen), anti-K48-linkage Specific Polyubiquitin (Cat: #8081, Cell Signaling Technology), anti-K63-linkage Specific Polyubiquitin (Cat: #5621, Cell Signaling Technology).

### Immunohistochemistry

A lung tissue microarray containing 98 cases of lung adenocarcinoma and paired adjacent non-cancerous tissue was purchased from Shanghai Outdo Biotech (HLugA180Su08). The incubated slides were then deparaffinized in xylene and rehydrated with graded alcohol. Next, antigens were retrieved using citrate buffer (pH 6.0). The samples were covered with 10% normal goat serum in phosphate buffered saline (PBS) for approximately 10 min at room temperature and then incubated with anti-TRIM15 (Catalog: PA5-40946, Invitrogen) or Nrf2 (Catalog: PA5-40946, Invitrogen) at 4 °C overnight. For immunohistochemical detection an HRP-Polymer Detection Kit (Abcam) followed by a DAB Substrate Kit (Abcam) were used, and slides were subsequently counterstained with haematoxylin. The staining intensity was scored as follows: 0 (0% to ≤ 25%), 1 (25% to ≤ 50%), 2 (50% to ≤ 75%), and 3 (> 75%). The 0 and 1 groups were defined as low expression, while 2 and 3 groups were defined as high expression.

### Co-immunoprecipitation assay

Co-IP assays were performed as described previously. Briefly, cells were harvested in IP lysis buffer (20 mM Tris pH 7.5, 150 mM NaCl, 1% TritonX-100, 1 mM ethylenediaminetetraacetic acid (EDTA), and protease inhibitors), incubated for 40 min on ice and centrifuged at 12,000×*g* at 4 °C for 10 min. Cell lysates were immunoprecipitated with antibodies against TRIM15 and Keap1 or negative control IgG and protein A/G agarose beads overnight with rotation. These beads were washed three times with lysis buffer. After separation by SDS-PAGE, the immunoprecipitates were subjected to Western blot analysis.

### Ubiquitination assay

To detect the ubiquitination levels of Keap1, cells were transfected with or without indicated plasmids for different experimental purpose and treated with 10 μM MG132 for 4 h to block proteasomal degradation. The cells were lysed and immunoprecipitated for 4 h at 4 °C with Protein G Magnetic beads loaded or bound with anti-HA antibodies according to immunoprecipitation assay described above. The immunoprecipitated proteins were subjected to immunoblotting analysis with antibody against Ubiquitin.

### CCK-8 cell proliferation assays

Cell viability was detected using the CCK8 assay (Dojindo, Tokyo, Japan). Briefly, the NSCLC cells stably transfected with shRNA were seeded into 96-well plates at a density of 4 × 10^3^ cells per well. After 48 h, 10 μL of the CCK8 solution was added into each well. After an additional 4 h incubation, absorbance at 450 nm was measured using a microplate reader.

### EdU incorporation assay

In the EdU assay, cells were plated in 12-well plates and transfected. After 48 h, EdU assay Kit (RiboBio Inc., Guangzhou, China) was performed to analyze cell proliferation per the manufacturer’s instructions. The cells were incubated with 10 μM EdU solution for 2 h and fixed with 4% paraformaldehyde, and the cells were washed 3 times with PBS and once with 0.5% TritonX-100. Next, the cell nuclei were stained with DAPI at a concentration of 1 µg/ml for 20 min. The proportion of the cells incorporated EdU was determined with fluorescence microscopy. After washing 3 times with PBS, we obtained images from a fluorescence microscope for further calculation of proliferation rates.

### Quantitative RT-PCR

Total RNA was isolated from the cells using TRIzol Reagent (Thermo Scientific, MA). qPCR analysis was performed using standard procedures on a StepOnePlus Real-Time PCR System (Applied Biosystems, CA). PCR reactions were performed in triplicate and the relative amount of cDNA was calculated by the comparative CT method using RPS21 as an endogenous control. RT-PCR was performed in at least three biological replicates. The Taqman probe for NQO1 (Cat: HP101580), PRDX1 (Cat: HP101099), TXN (Cat: HP100418), FTL (Cat: HP104808), and GAPDH (Cat: HP100003) were purchased from Sino Biological Inc. (Beijing, China). The primers for Human GCLC: 5′-ATGTGGACACCCGATGCAGTATT-3′ (forward) and 5′-TGTCTTGCTTGTAGTCAGGATGGTTT-3′ (reverse) and HMOX: 5′-AACAAGCAGAACCCAGTCTATGC-3′ (forward) and 5′-AGGTAGCGGGTATATGCGTGGGCC-3′ (reverse). Gene expression was normalized to housekeeping gene expression (GAPDH).

### Migration and invasion assays

Cells were allowed to form a confluent monolayer in a 24 well plate. The wound was created by scraping a conventional pipette tip across the monolayer. Cells were washed with PBS, cultured in serum and antibiotic free media at 37 °C, and photographed at 0 and 24 h.

For the in vitro invasion assays, cells in serum-free medium were seeded on a Matrigel-coated transwell insert (8 μm pore size, Corning, BD Biosciences). The lower chamber contained DMEM supplemented with 10% FBS as a chemoattractant. After incubation for 24 h, the inserts were fixed and stained for 30 min with 0.4% crystal violet dissolved in methyl alcohol, and the cell numbers were calculated by averaging the counts of three random fields.

### Plasmid constructs and RNA interference

TRIM15 (Cat: HG23822-UT), Flag-TRIM15, HA-Keap1 (Cat: HG11981-NY), Nrf2 (Cat: HG17384-ACR), and Myc-Nrf2 (Cat: MG56971-NM) expression plasmid was purchased from Sino Biological Inc. Scrambled, human TRIM15 and Nrf2 short hairpin RNAs (shRNAs) was obtained from Shanghai Genechem Co., Ltd. (Shanghai, China). To elucidate the role of TRIM15 as an E3 ligase, we generated an E3 ligase-defective TRIM15 by using the KOD-Plus-Mutagenesis kit (Toyobo, Cat: SMK-101) and verified by performing DNA sequencing. Plasmid transfection was performed using the Lipofectamine 3000 transfection reagent (Thermo Fisher, Cat. #L3000015) according to the manufacturer's instructions. After 48 h, biological and biochemical experiments were performed.

### Antioxidant response element (ARE) activity assays

The 8xARE-Luc construct was generated by cloning 8 copies of the antioxidant response element into the pGL3-promoter vector that contains the firefly luciferase gene. NSCLC cells were transfected with the 8xARE-Luc construct along with a renilla luciferase construct. 48 h after transfection, cells were subjected to overnight drug treatments and luciferase assays were performed using the Dual-Glo luciferase assay system. The firefly luciferase activity was, then, normalized to the renilla luciferase activity.

### Immunofluorescence confocal imaging

Cells were grown on Lab-Tek chamber slides, fixed with 4% paraformaldehyde in PBS for 30 min, and permeabilized with 0.1% Triton X-100 in PBS for 5 min. The slides were incubated with primary antibodies in blocking solution overnight at 4 °C in a humidified chamber. Subsequently, the glass slides were washed three times in PBS and incubated with Alexa Fluor 594-conjugated and Alexa Fluor 488-conjugated secondary antibodies and 4, 6-diamidino-2-phenylindole (DAPI) in blocking solution for 30 min at 37 °C in a humidified chamber. Images were acquired with a Leica confocal system.

### Xenograft transplantation experiments

For subcutaneous xenografting, H1299 or H1650 cells were injected subcutaneously into 6-week-old male BALB/c nude mice. Tumors were measured twice weekly using calipers and the volume determined using the formula: V = (S2 × L)/2, where V is the volume, S is the shortest diameter, and L is the longest diameter. The mice were euthanized on day 30, and the tumor size and weight were measured. For the lung metastasis studies, H1299 or H1650 cells were injected into 6-week-old male BALB/c nude mice via the tail vein. Mice were sacrificed one months later, and the metastatic nodules were counted. Metastatic tissues were analyzed by hematoxylin and eosin staining. All murine procedures were approved by the Institutional Animal Care and Use Committee at Wannan Medical College.

### Statistical analysis

Statistics were performed using GraphPad Prism 7 (Graph Pad Software Inc). The relationship of TRIM15 expression and clinicopathological parameters was evaluated with chi-squared test. The Kaplan–Meier method was used to analyse patient survival. Student's t tests were used to evaluate continuous variables between subgroups. *P* value of less than 0.05 was considered statistically significant. All group numbers and explanation of significant values are presented within the figure legends.

## Results

### TRIM15 is upregulated in NSCLC and associated with NSCLC progression

**Fig. 1:**
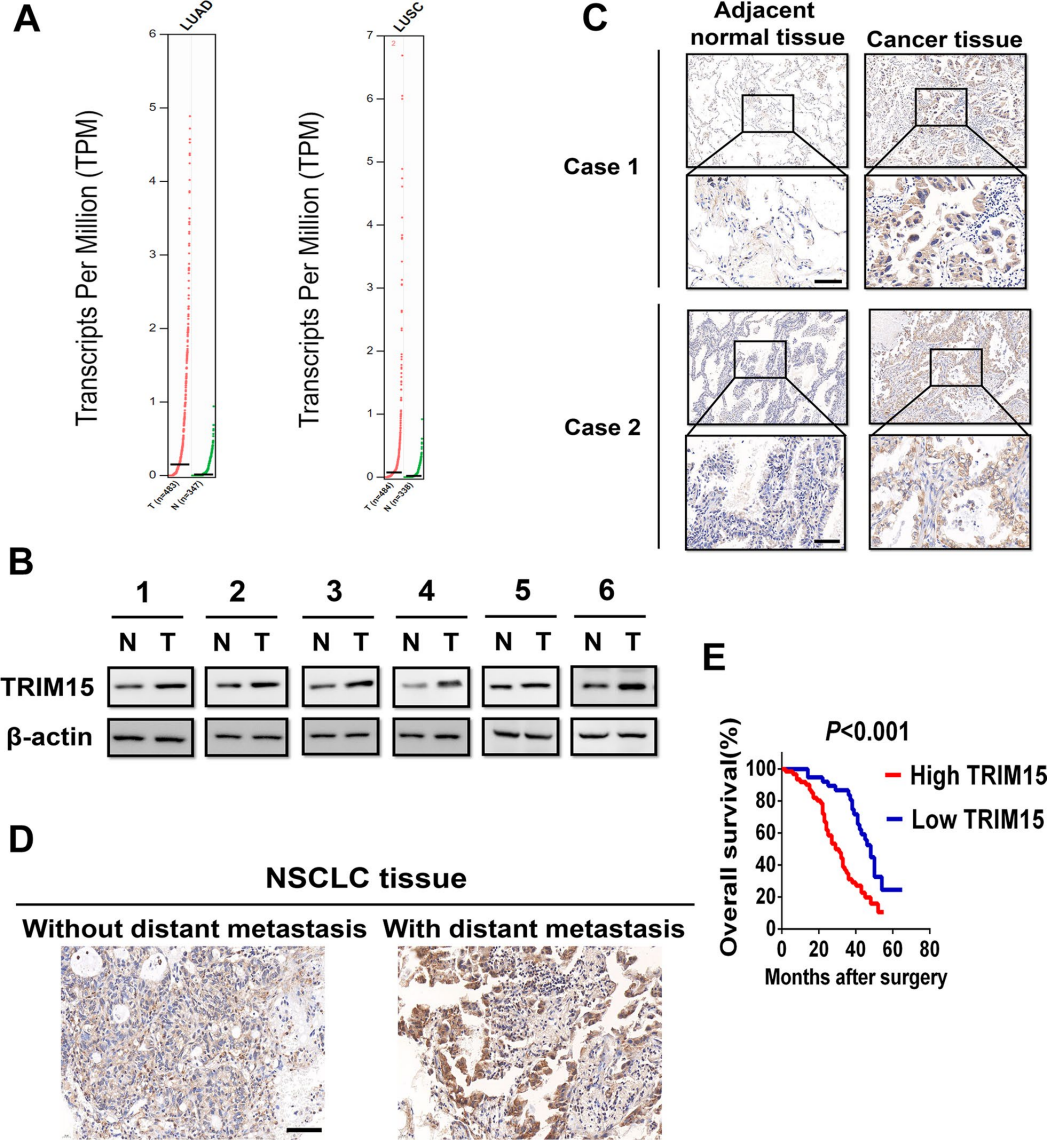
TRIM15 is upregulated in NSCLC and associated with NSCLC progression. **A** The gene expression of TRIM15 between NSCLC samples and normal lung tissue from GEPIA. LUAD (Lung adenocarcinoma), LUSC (Lung squamous cell carcinoma). **B** Western blot analysis of TRIM15 expression in NSCLC tissues compared with corresponding noncancerous lung tissues. **C** IHC analysis of TRIM15 expression in 2 NSCLC samples. Representative images are shown. Scale bar, 100 μm. **D** Representative IHC images of TRIM15 in NSCLC tissues with or without distant metastasis. Scale bar, 200 μm. **E** OS analysis of patients with NSCLC stratified by the TRIM15 expression level in 98 samples. Patients with high-expression levels of TRIM15 had shorter OS times than patients with low expression levels. **P < 0.01

To examine the expression pattern of TRIM15 in NSCLC, we analysed the level of TRIM15 mRNA using the GEPIA online tool (http://gepia.cancer-pku.cn/) in which the data from The Cancer Genome Atlas (TCGA) database and GTEx data. TRIM15 mRNA level was significantly higher in NSCLC samples than in normal tissue (Fig. 1A). To determine the significance of TRIM15 in NSCLC development, we first examined TRIM15 expression in 6 NSCLC samples using western blot analysis. TRIM15 was significantly upregulated in NSCLC tissues compared with adjacent non-cancerous lung tissues (Fig. 1B). Immunohistochemical staining further confirmed that TRIM15 was overexpressed in tumor tissues than matched surrounding tissues (Fig. 1C). Further analysis showed TRIM15 levels to be markedly higher in the NSCLC tissues with distant metastasis than in NSCLC tissues without distant metastasis (Fig. 1D).

We further examined the expression of TRIM15 by immunohistochemical staining on a human lung adenocarcinoma tissue array containing 98 tissue samples, whose clinicopathological features and complete follow-up data was performed. Correlation analysis revealed that high expression of TRIM15 in NSCLC tissues was significantly associated with a more aggressive tumor phenotype (Additional file 1: Table S1). Kaplan–Meier analysis indicated that patients with high TRIM15 expression had a significantly lower survival rate than those with low TRIM15 expression (Fig. 1E). Taken together, these findings indicated a strong correlation between high expression of TRIM15 and lung cancer malignancy, suggesting that TRIM15 may play a role in lung cancer development and progression.

### TRIM15 promotes NSCLC proliferation and metastasis

**Fig. 2:**
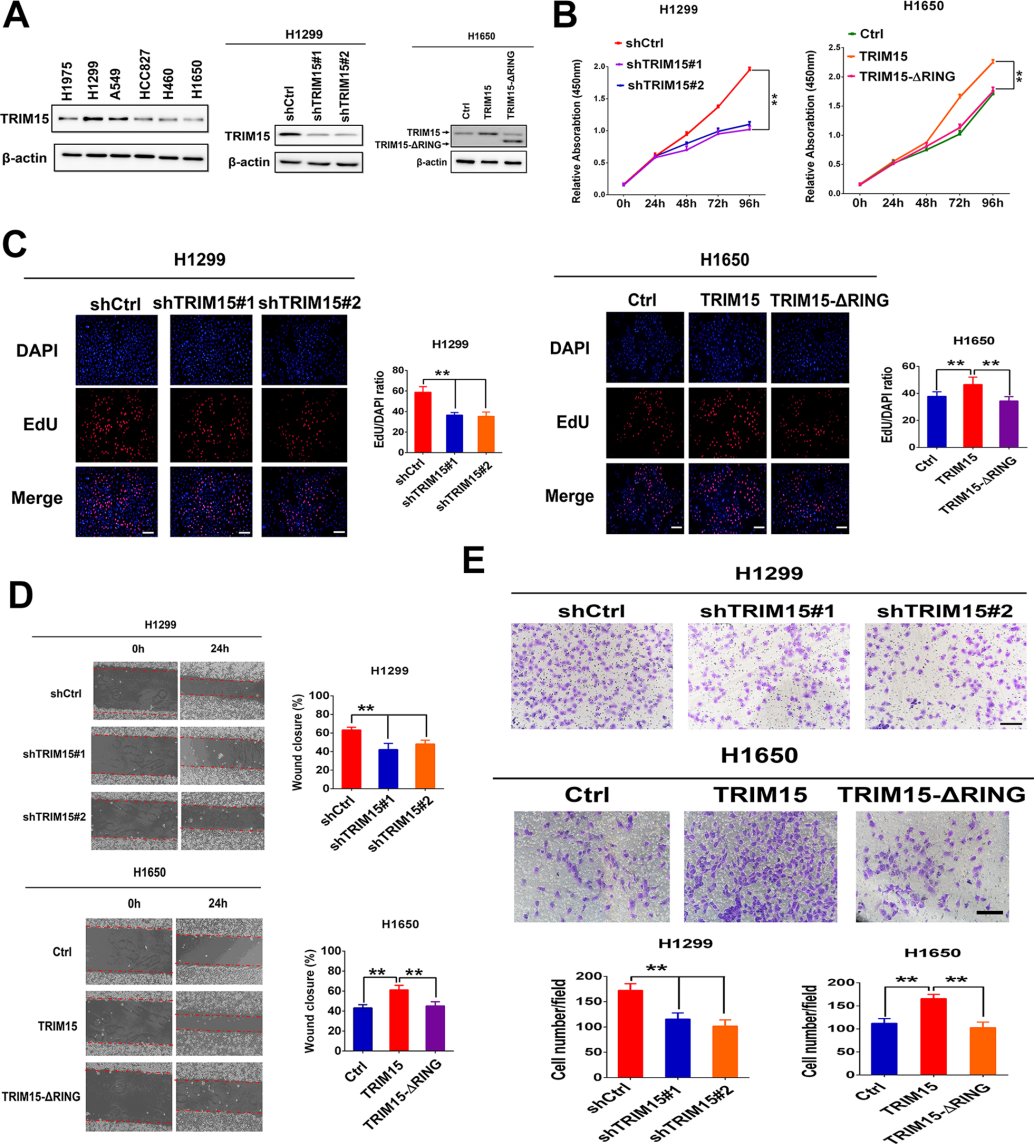
Roles of TRIM15 in promoting NSCLC growth and metastasis. **A** Expression levels of TRIM15 in the indicated lung cancer cell lines were analysed by western blotting. Confirmation of TRIM15 knockdown in H1299. H1650 cells were transfected with control plasmids or vector expressing TRIM15 or TRIM15-ΔRING. Immunoblot detection of TRIM15 and TRIM15-ΔRING expression in H1650 cells. **B**, **C** The effect of TRIM15 knockdown and overexpression on NSCLC cells proliferation was assessed by CCK8 and EdU-incorporation assay. **D**, **E** The effects of TRIM15 loss- or gain-of-function on migration (**D**) and invasion (**E**) of NSCLC cells. Knockdown of TRIM15 resulted in significant inhibited migration (**D**) and invasion (**E**) of H1299 cells. TRIM15 upregulation significantly increased cancer cell migration (**D**) and invasion (**E**), while the TRIM15-ΔRING lost this ability. Statistical analyses were performed by two-tailed unpaired Student’s *t*-test. **P < 0.01

The above data showed that TRIM15 positively correlates with a more aggressive tumor phenotype. Accordingly, we investigated the functions of TRIM15 in NSCLC cell lines using in vitro assays. To evaluate the effects of TRIM15 on in vitro cell proliferation, migration, and invasion, we first investigated the endogenous TRIM15 levels of different NSCLC cell lines (Fig. 2A). NSCLC cells were selected for loss- or gain-of-function studies due to their high or low endogenous TRIM15 levels. First, we investigated the effects of knocking down TRIM15 by generating two stable shRNA expressing H1299 cells, which displayed relatively higher TRIM15 expression among all tested NSCLC cells cell lines (Fig. 2A). CCK8 and EdU assays showed TRIM15 knockdown induced by shTRIM15 indeed resulted in a considerable inhibitory effect on in vitro proliferation of H1299 cells (Fig. 2B, C). Furthermore, silencing TRIM15 also induced potent suppression on migration and invasion of H1299 cells (Fig. 2D, E).

To assess the enzymatic activity of TRIM15 in NSCLC cells, we generated an E3 ligase-defective TRIM15 mutant (TRIM15-∆RING), in which the N-terminal RING domain was deleted and the potential E3 ubiquitin ligase activity was deprived. Wild-type or TRIM15-∆RING was stably overexpressed in H1650 cells. Consistent with these findings, exogenous TRIM15 expression in H1650 cells significantly enhanced its proliferation, migration, and invasion capacity compared with control cells, while the TRIM15-∆RING showed no obvious oncogenic functions (Fig. 2B–E). Therefore, the functions of TRIM15 in the regulation of cell proliferation, migration, and invasion might be dependent on its E3 ligase. Taken together, these gain- and loss-of-functional studies demonstrated that TRIM15 plays important roles in promoting NSCLC growth and metastasis.

### E3 ligase TRIM15 interacts with Keap1

**Fig. 3:**
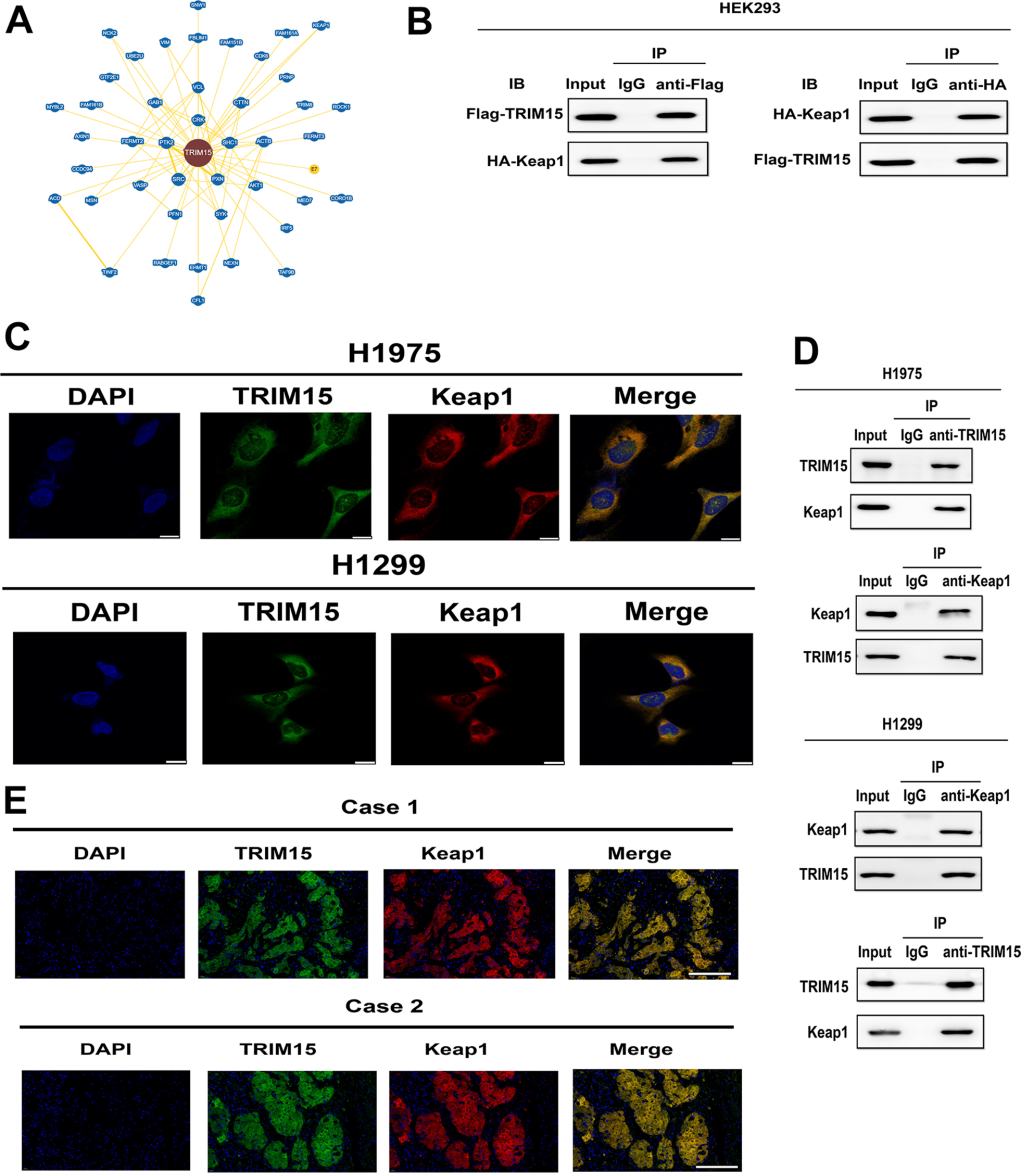
TRIM15 associates with Keap1. **A** Network graph representation of interaction from the BioGRID for TRIM15. Users can select the ‘Network’ tab from the ‘Switch View’ menu to view interactions data when available. **B** Immunoblot detection of the indicated proteins in a Co-IP assay performed in HEK293 cells. **C** Immunofluorescence colocalization of TRIM15 with Keap1 in H1299 and H1975 was assessed by rabbit anti-TRIM15 detected with anti-rabbit IgG-Alexa Fluor 488 (green fluorescence), and detection of Keap1 with mouse anti-Keap1 detected with anti-mouse IgG Alexa 594 (red fluorescence). The colocalization of TRIM15 and Keap1 is illustrated by overlay of the images, illustrated by yellow fluorescence. **D** Immunoprecipitation assay revealing the interaction between TRIM15 with Keap1 in H1299 and H1975 cells. **E** Co-localization of TRIM15 (green) and Keap1 (red) in 2 NSCLC tissues from two patients by immunofluorescent confocal microscopy

There is increasing evidence that some TRIM proteins act as E3 ubiquitin ligases in specific ubiquitin-mediated protein degradation pathways. Structure–function evaluations also demonstrated that the tumor-promoting functions of TRIM15 depend on its E3 ubiquitin ligase. These functional characteristics enabled us to further address which protein TRIM15 binds to and how this interaction affects the physiological function of the target protein. Protein-fragment complementation assay showed that TRIM15 could interact with Keap1 [20] (Fig. 3A). Hence, we evaluated the potential interaction between the two proteins. To this end, co-immunoprecipitations in human embryonic kidney (HEK293) cells expressing Flag-TRIM15 and HA-Keap1 proteins were performed. Indeed, TRIM15 and Keap1 proteins were reciprocally coimmunoprecipitated (Fig. 3B). Moreover, immunofluourescence evaluations of H1299 and H1975 cells demonstrated that TRIM15 and Keap1 frequently colocalize in these cells (Fig. 3C). Furthermore, endogenous TRIM15 also bound endogenous Keap1 from the H1299 and H1975 cells (Fig. 3D). We also demonstrated that the interaction occurred in human primary NSCLC tissues as well (Fig. 3E). Collectively, these findings established that TRIM15 physically interacts with Keap1.

### TRIM15 promotes Keap1 ubiquitination and degradation

**Fig. 4:**
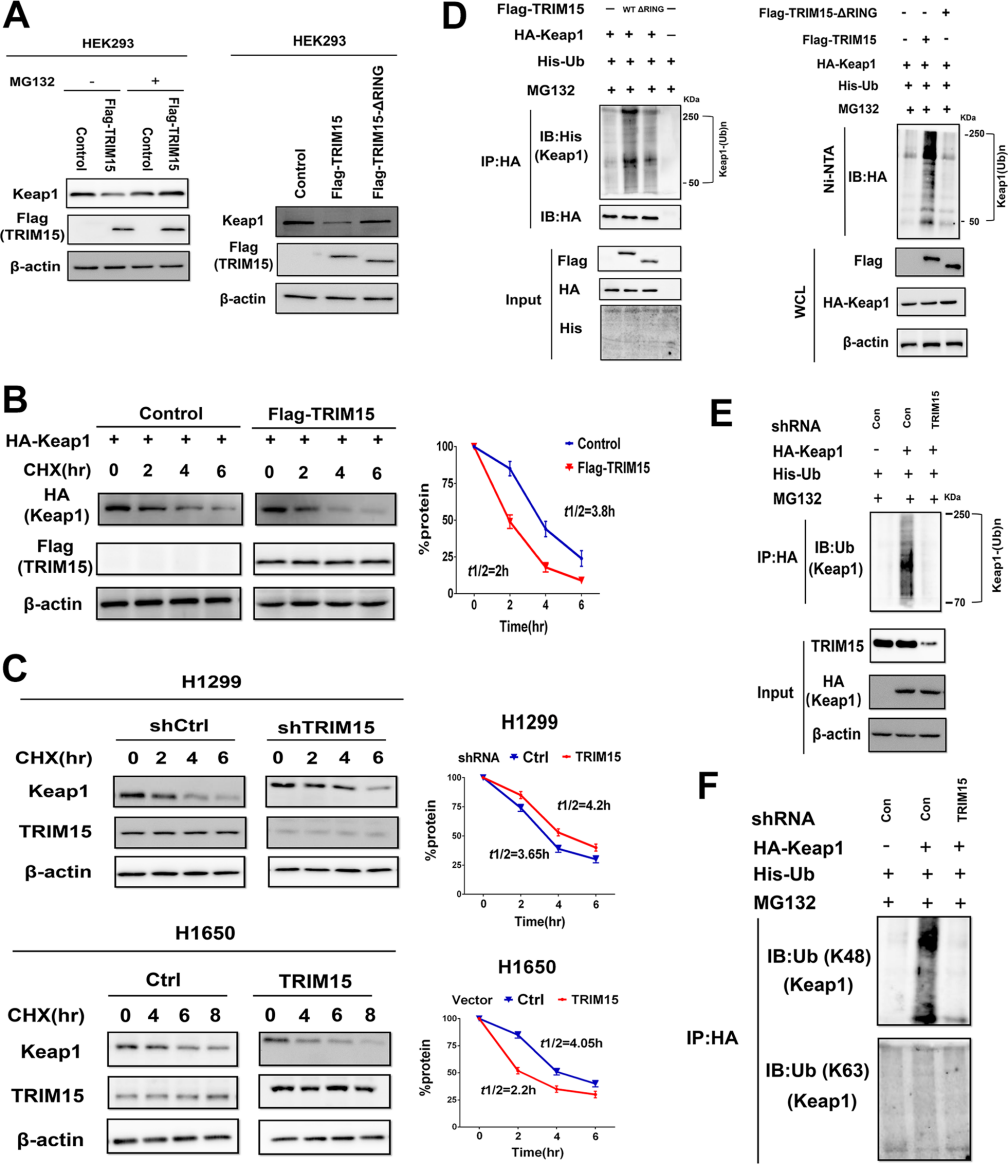
TRIM15 promotes Keap1 ubiquitination and degradation. **A** Immunoblot analysis of Keap1, Flag-TRIM15, and β-actin in HEK293 cells transfected with expression vector for Flag-TRIM15 or with empty vector, with or without treatment of 10 μM MG132. Mutations of Flag-TRIM15-ΔRING impaired the ability of TRIM15 to degrade Keap1 protein in HEK293 cells. **B** HEK293 cells were transfected with the indicated plasmids. The cells were treated with 50 μg/ml CHX for indicated time periods. Densitometry analysis performed on corresponding immunoblots to assess Keap1 half-life in the indicated conditions. **C** H1299 or H1650 cells were transfected with the indicated plasmids, treated with 50 μg/ml CHX, harvested at different time points, and then immunoblotted with using the indicated antibodies. Densitometry analysis performed on corresponding immunoblots to assess Keap1 half-life in the indicated conditions. **D** HEK293 cells were transfected with Flag-TRIM15, Flag-TRIM15-ΔRING, HA-Keap1, and His-ubiquitin plasmids, and the cell lysates were subjected to immunoprecipitation using anti-HA antibodies (left panel) or Ni–NTA pull-down (right panel) under denaturing conditions, followed by immunoblotting with the indicated antibodies. Overexpression of wild-type but not the mutant TRIM15-ΔRING promoted ubiquitination of Keap1. **E** Knockdown of endogenous TRIM15 decreased the ubiquitination of HA-Keap1 in HEK293 cells analyzed by in vitro ubiquitination assays. **F** Cell lysates prepared in **D** were immunoprecipitated with anti-HA. knockdown of TRIM15 inhibits K48-linked but not K63-linked ubiquitination of HA-Keap1

Because TRIM15 is an E3 ubiquitin ligase, we next determined whether Keap1 is the ubiquitination target of TRIM15. We expressed Flag-TRIM15 or vector control in HEK293 cells with or without treatment of MG132 and analyzed the expression of Keap1 and TRIM15. As results, TRIM15 could significantly degrade Keap1, compared with the vector control (Fig. 4A). Additionally, the treatment of MG132 could rescue the expression of Keap1 (Fig. 4A). We further investigated whether the above-mentioned TRIM15-ΔRING mutant can impair the ability of TRIM15 to degrade Keap1 in cells. In accord with these findings, TRIM15-∆RING mutants largely abolished the ability of TRIM15 to degrade Keap1 in HEK293 cells (Fig. 4A). We next evaluated the impact of TRIM15 on Keap1 stability. To assess the effect of TRIM15 on Keap1 half-life, Keap1 levels were assessed by immunoblot following overexpression of Flag-TRIM15 in HEK293 cells. We further found ectopic expression of Flag-TRIM15 shortened the half-life of Keap1 (Fig. 4B). Importantly, knockdown of endogenous TRIM15 increased the half-life of Keap1 protein in H1299 cells. In contrast, exogenous upregulation of TRIM15 expression shortened Keap1 half-life in H1650 cells (Fig. 4C). Collectively, these data indicated that that TRIM15 probably mediates Keap1 degradation by ubiquitin–proteasome pathway.

To further investigate whether TRIM15 promotes Keap1 degradation through ubiquitination, we performed in vitro ubiquitination assay. HEK293 cells were transfected with plasmids encoding Flag-TRIM15 or mutant Flag-TRIM15-∆RING and HA-Keap1 and His-ubiquitin, respectively. Cells with Flag-TRIM15 expression displayed increased ubiquitination of HA-Keap1 compared with cells transfected with the control vector (Fig. 4D). Notably, Keap1 ubiquitination was not observed in cells expressing the TRIM15-ΔRING mutant suggesting that TRIM15 potentiated the ubiquitination of Keap1 through its E3 ligase activity (Fig. 4D). In keeping with this finding, knockdown of endogenous TRIM15 decreased HA-Keap1 ubiquitination in HEK293 cells (Fig. 4E). Furthermore, silencing TRIM15 inhibited K48-linked but not K63-linked ubiquitination of Keap1 (Fig. 4F). A K48-linked polyubiquitin chain is associated with proteins marked for degradation by the 26S proteasome. Thus, these results indicated that TRIM15 promotes the degradation of Keap1 by heightening K48-linked ubiquitination of Keap1. When viewed in combination, these results demonstrated that the E3 ubiquitin ligase TRIM15 downregulates Keap1 through ubiquitination and proteasomal degradation.

### TRIM15 stabilizes Nrf2 through binding with Keap1

**Fig. 5:**
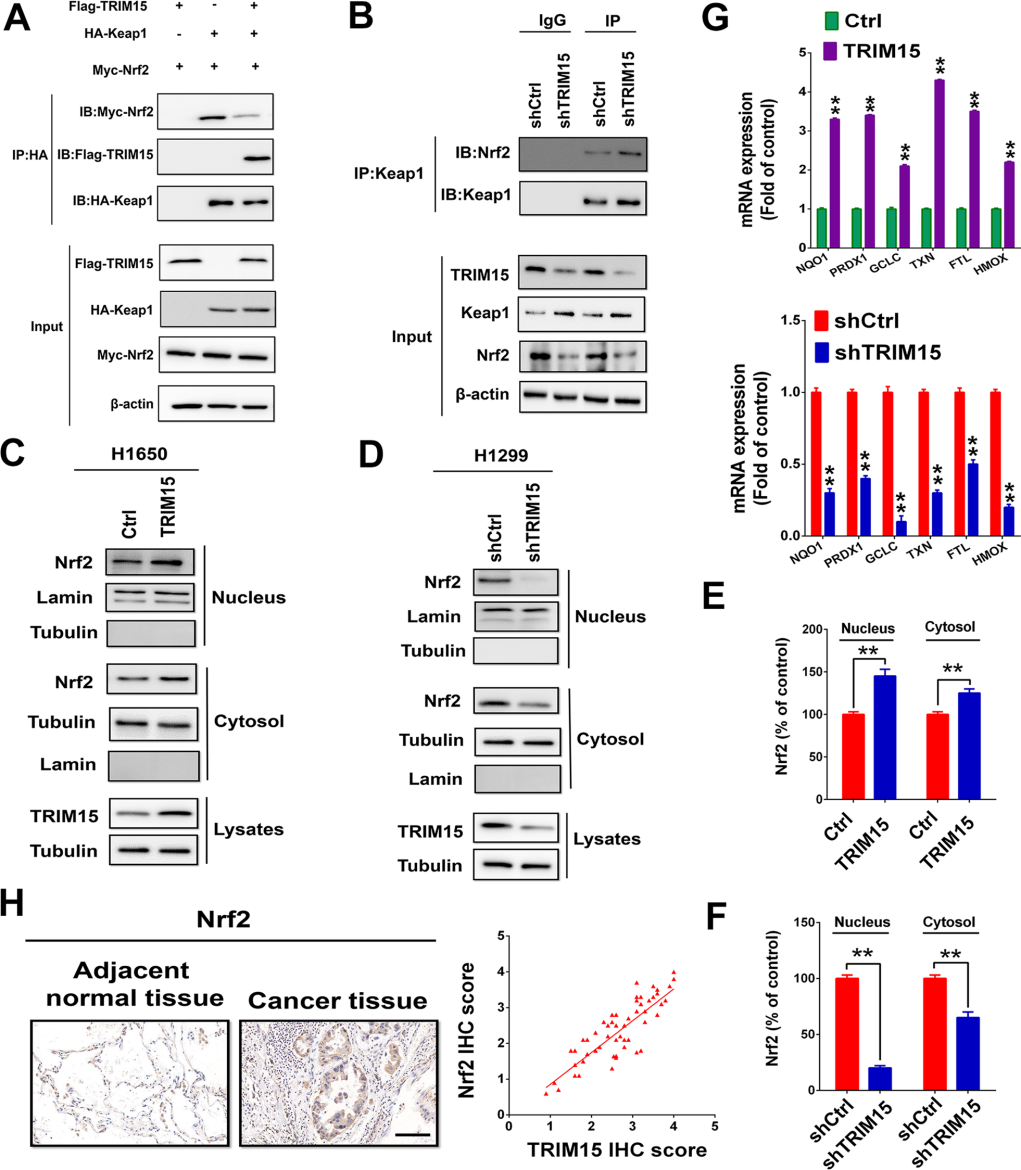
TRIM15 stabilizes Nrf2 through binding with Keap1. **A** HEK293 cells transfected with Flag-TRIM15, HA-Keap1, and Myc-Nrf2 were subjected to immunoprecipitation with HA antibody. Lysates were analyzed by western blotting. **B** TRIM15 reduced the interaction between Nrf2 and Keap1. Cell lysates were immunoprecipitated with an anti-Keap1 antibody and blotted with an anti-Nrf2 antibody. **C**–**F** Subcellular fractionation was used to isolate cytoplasmic and nuclear proteins, and immunoblotting was performed to examine the localization of Nrf2 following the downregulation or overexpression of TRIM15. Nuclear and cytoplasmic levels of Nrf2 are quantified. **G** Effect of TRIM15 knockdown (H1299 cells) or overexpression (H1650 cells) on the mRNA expression of the Nrf2-regulated genes. NAD(P)H quinone dehydrogenase1(NOQ1), thioredoxin (TXN), peroxiredoxin 1(PRDX1), hemoxygenase 1(HMOX1), glutamate-cysteine ligase catalytic subunit (GCLC), glutathione S-transferase μ1(GSTM1), glutathione S-transferase μ3(GSTM3), ferritin light chain (FTL). **H** Representative IHC staining images of Nrf2 in the same set of NSCLC tissue slices. Correlation analysis of TRIM15 and Nrf2 expression in NSCLC samples. Spearman correlation coefficients are shown. Scale bars, 100 μm. Statistical analyses were performed by two-tailed unpaired Student’s *t*-test. **P < 0.01

Keap1 is well known to act as a substrate adaptor to bring Nrf2 into the Cul3-dependent E3 ubiquitin ligase complex, resulting in the rapid proteasome-mediated degradation of Nrf2 [6]. We thus investigated whether TRIM15 regulates Nrf2 protein stability due to TRIM15-dependent degradation of Keap1. To determine whether TRIM15 overexpression might inhibit the interaction of Keap1 and Nrf2, HA-Keap1, Myc-Nrf2, and Flag-TRIM15 were co-transfected into HEK293 cells. In co-immunoprecipitation assays, we observed that upregulated TRIM15 competitively inhibited the Keap1-Nrf2 interaction and subsequently stabilized Myc-Nrf2 levels (Fig. 5A). To further verify this finding, immunoprecipitation assays were also performed in H1299 cells expressing control or TRIM15shRNA. Consistent with this finding, TRIM15 knockdown significantly increased Keap1-Nrf2 binding and subsequently decreased protein abundance of Nrf2 in H1299 cells (Fig. 5B). The results showed that TRIM15 competitively binds to Keap1, thus inhibiting the interaction of Keap1 and Nrf2 and subsequent degradation of Nrf2.

**Fig. 6:**
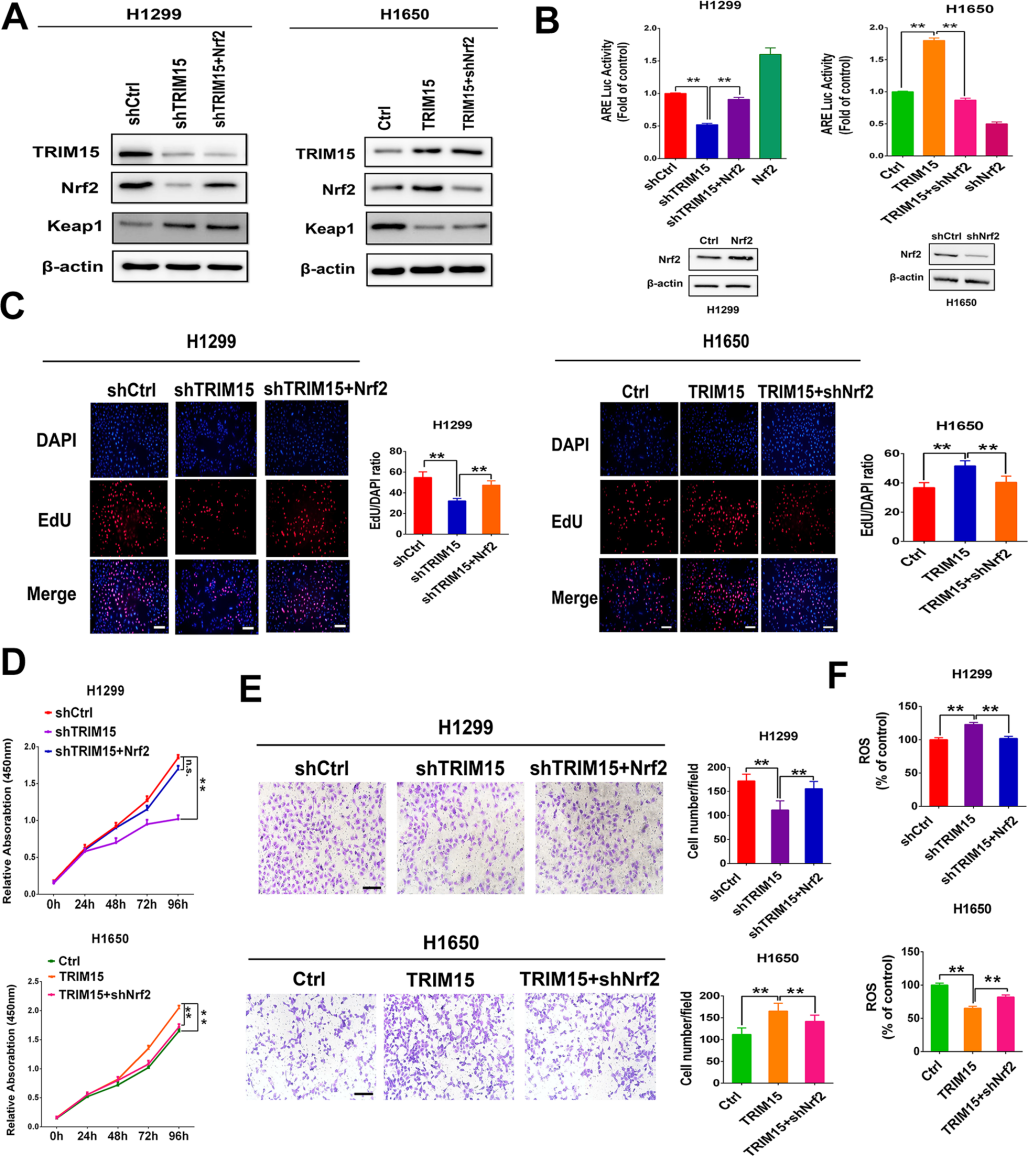
TRIM15-mediated Nrf2 signaling regulates growth and invasion in NSCLC cells in vitro. **A** Western blot analyses of TRIM15, Nrf2, Keap1, and Nrf2 target NQO1 in H1299 cells with TRIM15 knockdown with or without subsequent Nrf2 overexpression and H1650 cells overexpressing TRIM15 with or without subsequent knockdown of Nrf2. **B** ARE Luc reporter activity assessed in H1299 cells expressing shTRIM15, sh TRIM15 + Nrf2 or H1650 cells overexpressing TRIM15 with or without subsequent knockdown of Nrf2. Up-regulating of Nrf2 expression in H1299 cells or down-regulating of Nrf2 expression in H1650 cells was set as a control. **C**–**F** Cell proliferation (**C**, **D**), invasion (**E**) and ROS formation (**F**) in H1299 cells with or without shTRIM15 and Nrf2 rescue or in H1650 cells with or without TRIM15 overexpression and shNrf2 rescue. Statistical analyses were performed by two-tailed unpaired Student’s *t*-test. **P < 0.01

As Nrf2 is widely accepted to be a key transcription factor responsible for regulating the antioxidant defense systems [4], we questioned whether TRIM15 influences antioxidant protein expression through regulating the Nrf2 signaling pathways. Consistent with upregulated total Nrf2 levels, we observed that high TRIM15 levels was positively associated with the nuclear levels of Nrf2 in H1650 cells (Fig. 5C, E). In contrast, depletion of TRIM15 leads to markedly decrease Nrf2 nuclear translocation in H1299 cells (Fig. 5D, F). Furthermore, modulation of TRIM15 levels caused a corresponding alteration in expression of Nrf2-regulated genes, such as NQO1, PRDX1, HMOX1, GCLC, TXN1, and FTL1. To further detect whether TRIM15 was sufficient to enhance Nrf2-antioxidant response elements (ARE) signaling, ARE luciferase reporter assay was performed. These results showed that the functions of TRIM15 in the regulation of ARE activity might be dependent on Nrf2 levels (Figs. 5G, 6A, B). Moreover, TRIM15 expression was positively associated with Nrf2 expression in NSCLC specimens (Fig. 5H). Taken together, these results indicated that TRIM15 regulates the antioxidant system by stabilizing Nrf2 protein levels and subsequently upregulating Nrf2-ARE signaling.

### Nrf2 is required for TRIM15 to facilitate tumor cell growth and invasion

The above results demonstrated that TRIM15 regulates growth and invasion in NSCLC cells. To further assess whether TRIM15 promotes tumor cell proliferation and metastasis dependent on Nrf2 signaling, we first silenced TRIM15 in the absence or presence of Nrf2 rescue. In H1299 cells, TRIM15 knockdown significantly suppressed proliferation, migration, and invasion of tumor cells, and increased ROS levels, and these effects were recovered by Nrf2 rescue (Fig. 6C–F). Conversely, TRIM15 overexpression remarkably promoted cell proliferation and invasion, and reduced ROS levels in H1650 cells. These effects were blocked, at least in part, by Nrf2 knockdown (Fig. 6C–F). Overall, these data indicated that TRIM15-mediated regulation of Nrf2 and ROS is critical for NSCLC cell growth and invasion.

### TRIM15 activates Nrf2 signaling pathway regulates growth and metastasis in vivo

**Fig. 7:**
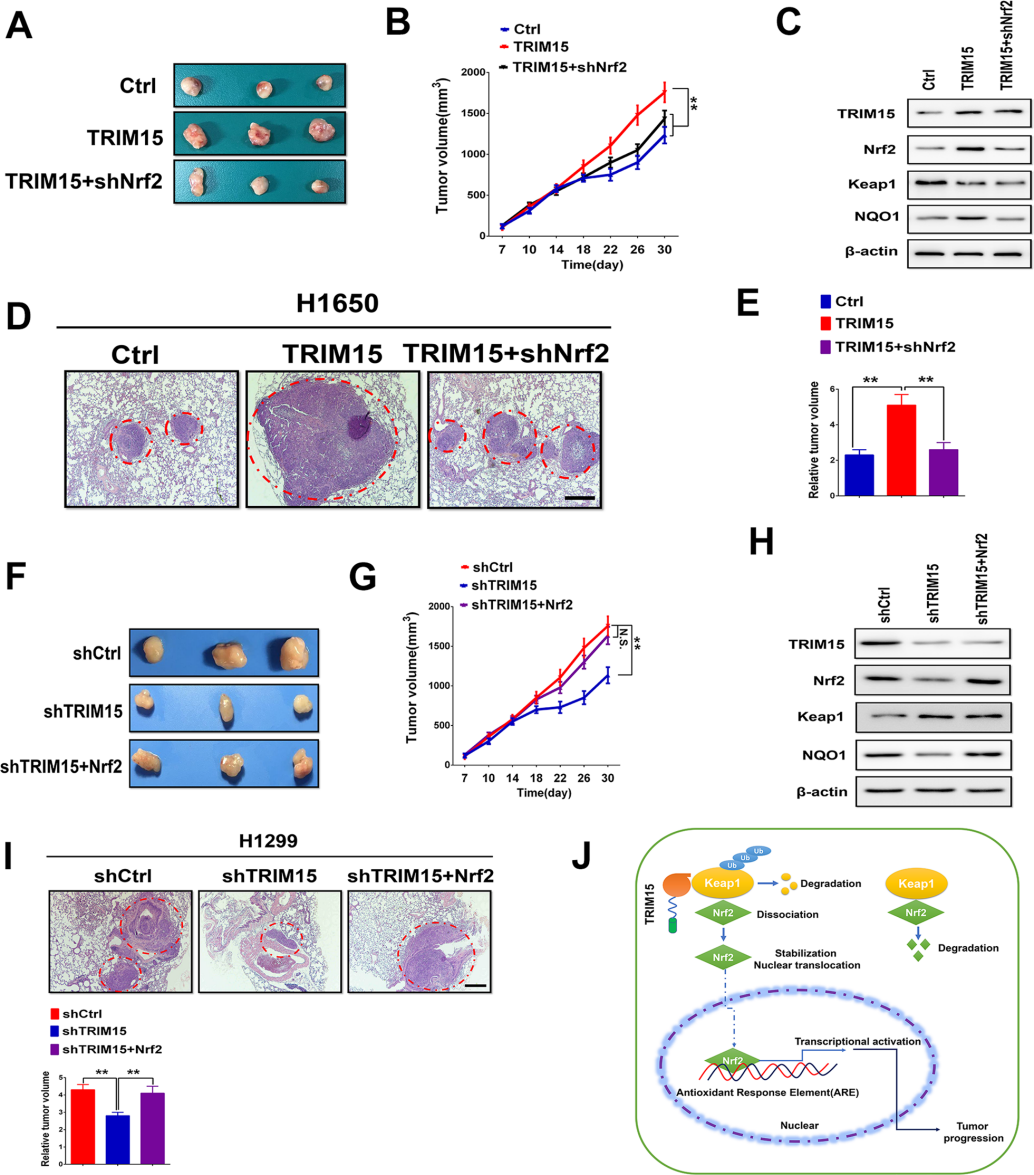
TRIM15 mediated increase in Nrf2 regulates growth and invasion in vivo. **A** Nude mice were randomized into three groups and subcutaneously injected with H1650 cells that had been transfected with control (empty vector), TRIM15, or TRIM15 + shNrf2 plasmids. Tumors formed in nude mice were collected 30 days after grafting, and the tumor weight were measured. **B** Measurement of tumor volume in experimental groups over time. **C** Western blotting analysis was performed to evaluate the levels of TRIM15, Nrf2, Keap1, and NQO1 in harvested tumors. **D**, **E** Up-regulation of TRIM15 significantly promoted lung metastasis in H1650 xenograft nude mice models, whereas the suppression of Nrf2 prevented the tumor metastasis of TRIM15 overexpressing cells. Representative pictures of the lung metastases in nude mice by H&E staining. Quantification of lung metastases in all groups. Scale bar: 200 μm. **F**, **G** A representative image of tumor growth in nude mice subcutaneously inoculated with H1299 cells tranfected with shCtrl, shTRIM15 or shTRIM15 + Nrf2 plasmids. Tumor volumes were measured on the indicated days. **H** Western blotting analysis was performed to evaluate the levels of TRIM15, Nrf2, Keap1, and NQO1 in xenograft tumors. **I** Representative pictures of the lung metastases in nude mice by H&E staining. Quantification of lung metastases in all groups. Scale bar: 200 μm. **J** TRIM15 was significantly upregulated in NSCLC and that increased TRIM15 was associated with poor survival. TRIM15 promoted tumor proliferation and metastasis by activating Nrf2 signaling. Furthermore, TRIM15 regulated Nrf2 activity by modulating Keap1 and inducing its ubiquitination and degradation in NSCLC cells. Activation of Nrf2 facilitated tumor cell proliferation and invasion. N.S. represents no significant. Statistical analyses were performed by two-tailed unpaired Student’s *t*-test. **P < 0.01

To confirm these findings, we further examined the effects of TRIM15 expression on in vivo tumor growth and metastasis of NSCLC cells. In subcutaneous implantation nude mice models, overexpression of TRIM15 significantly promoted tumor growth compared with vector controls, and this effect was blocked when TRIM15 was co-expressed with shNrf2 (Fig. 7A, B). Moreover, up-regulated of TRIM15 increased Nrf2 and the target gene NQO1 expression in subcutaneous tumor tissues, an observation not seen with co-expression of TRIM15 with Nrf2shRNA (Fig. 7C). Moreover, TRIM15 also promoted lung metastasis in mice in a Nrf2-dependent manner (Fig. 7D, E). These results indicated that TRIM15 regulates the tumor growth and metastasis by stabilizing Nrf2 protein levels.

In a set of complementary experiments, we clarified the role of TRIM15 knockdown on tumor growth in vivo. H1299 cells with stable silencing of TRIM15 or control shRNA with or without overexpression of Nrf2 were injected subcutaneously into athymic mice to form subcutaneous tumors. As shown in Fig. 7F, G, downregulation of TRIM15 significantly inhibited tumor growth, whereas up-regulation of Nrf2 could promote the tumor growth of TRIM15 knockdown cells. Moreover, silencing of TRIM15 significantly reduced Nrf2 levels and NQO1 expression in vivo (Fig. 7H). Consistent with the aforementioned results, TRIM15 depletion also inhibited lung metastases in mice, whereas the metastatic capacity was increased in TRIM15-knockdown cells subjected to overexpression of Nrf2 (Fig. 7I). Collectively, these results indicated that the ability of TRIM15 to promote NSCLC development depends on the Nrf2 signaling pathway.

## Discussion

Protein ubiquitination is a critical regulator of cellular homeostasis. Aberrations in the addition or removal of ubiquitin can result in the development of cancer and key components of the ubiquitination machinery serve as oncogenes [21, 22]. In this study, we demonstrated that TRIM15 was upregulated in NSCLC and promoted cell proliferation and metastasis. Furthermore, the tumor-promoting functions of TRIM15 depended on its E3 ubiquitin ligase. This notion is supported by the TRIM15-ΔR mutant showed no obvious oncogenic functions. Mechanistically, TRIM15 directly ubiquitinated and degraded Keap1 through its ubiquitin E3 ubiquitin ligase, leading to the activation Nrf2 pathway. Activation of Nrf2 resulted in bolstering antioxidant response and tumor progression. To our knowledge, our research is the first to present the molecular mechanisms of TRIM15 in NSCLC.

TRIM proteins are involved in a broad range of oncogenic processes including transcriptional regulation, cell proliferation, apoptosis, DNA repair, and metastasis. Similar to many TRIM family members, TRIM15 contains a RING domain, a B-box type 2 zinc finger domain, and a coiled-coil domain [23]. Evidence suggests that TRIM15 is a potential diagnostic marker of gastric cancer that affects DNA methylation patterns [24]. Additionally, TRIM15 promotes the invasion and metastasis of pancreatic cancer cells by mediating APOA1 ubiquitination and degradation [19]. In this study, we demonstrated that TRIM15 upregulation was related to poor prognoses in NSCLC. Supporting a role of TRIM15 in tumorigenesis, TRIM15 depletion in H1299 cell line remarkably impaired tumor cell growth in vitro and in vivo. Remarkably, expression of WT but not mutant TRIM15 significantly increased cancer cell proliferation and invasion. These data provided strong evidence that the TRIM15 is a potential oncogene that drives tumorigenesis. Given that the oncogenic functions of TRIM15 rely on its enzymatic activity, we speculated that TRIM15 as E3 ubiquitin ligases exerts its function by interacting with certain key oncogenes or regulating tumor-promoting pathways. Consistent with our speculation, both in vitro and in vivo assays illustrated that upregulation of TRIM15 enhanced polyubiquitination of Keap1 and promoted its proteasomal degradation, and ultimately promoted the proliferation and metastasis of NSCLC. Consistent with these findings, we herein show that TRIM15 regulated the antioxidant system by stabilizing Nrf2 protein levels and subsequently upregulating Nrf2-ARE signaling. This finding not only further confirms the direct modulation of TRIM15 on Keap1, but also identifies TRIM15 as a potentially promising intervention target for NSCLC.

Redox status imbalance commonly appears in cancer [25]. During lung tumorigenesis, tumor cells exhibit permanent high ROS levels due to the oncogene activation, increased metabolic rates, hypoxia, mitochondrial and/or peroxisomal dysfunction as well as anchorage-independent growth [25, 26]. In this context, Nrf2 plays a key role acting as a major regulator of the antioxidant response [27]. Activation of the Nrf2 pathway could be advantageous to protect tumor cells from oxidative stress. To maintain oxidative homeostasis, approximately 30% of NSCLCs increase the transcription of antioxidant genes by acquiring either stabilizing mutations in Nrf2 or by selecting for inactivating mutations in its negative regulator, Keap1 [27–30]. It is well acknowledged that Nrf2 activation plays a critical role in various human cancers, such as hepatocellular carcinomas, lung cancer, and gallbladder cancer [31–33].Under basal conditions Nrf2 is polyubiquitinated by the Keap1-Cul3-E3 ligase and degraded by the 26S-proteasome. In response to oxidative stress, the Keap1-Nrf2 binding is inhibited and, consequently, Nrf2 is stabilized. Moreover, in a Kras-driven lung adenocarcinoma, Keap1 loss leads to the appearance of high-grade invasive adenocarcinomas that are typically associated with increased metastases [34, 35]. Similarly, loss of Keap1 in lung adenocarcinoma patients is associated with high-grade and late-stage disease and shortened survival, which might be caused by an increased rate of metastasis in these patients [35]. In this study, we provided evidence that TRIM15 represented an additional critical regulator of Nrf2 expression levels in NSCLC. TRIM15 mediated Nrf2 protein stabilization by enhancing Keap1 ubiquitination and degradation. Furthermore, TRIM15 competitively bound to Keap1, thereby inhibiting the Keap1-Nrf2 interaction. Consistently, Nrf2 is required for TRIM15 to facilitate tumor cell proliferation and metastasis in vitro and in vivo. We also observed upregulation of TRIM15 expression is positively associated with Nrf2 expression in NSCLC specimens. These findings demonstrated that TRIM15 promotes the proliferation and metastasis of NSCLC via stabilizing Nrf2 expression.

Our present study provides conclusive evidence that TRIM15 was significantly upregulated in NSCLC and that increased TRIM15 was associated with poor survival. TRIM15 directly targeted Keap1 by ubiquitination and degradation, promoting Nrf2 stabilization, and ultimately an increased proliferation and invasion of cancer cells. Therefore, it is of great interest to develop therapeutic approaches targeting the TRIM15-Keap1 interaction may offer a new therapeutic opportunity to block NSCLC growth and metastasis.

## Supplementary Information

**Additional file 1: Table S1**. Analysis of association between TRIM15 expression and clinicopathological parameters in NSCLC.

## Data Availability

The data presented in this manuscript are available upon reasonable request from the corresponding author.

## Abbreviations

NSCLC: Non-small cell lung cancer
TRIM: Tripartite motif
Nrf2: Nuclear factor, erythroid 2-like transcription factor 2
Keap1: Kelch-like ECH-associated protein 1
ARE: Antioxidant response elements
ROS: Reactive oxygen species
