# Supplementary material for: E3 ligase TRIM15 facilitates non-small cell lung cancer progression through mediating Keap1-Nrf2 signaling pathway
Source: Cell Commun Signal. 2022 May 9;20:62. doi: 10.1186/s12964-022-00875-7 (PMC9082862; doi:10.1186/s12964-022-00875-7)
Supplement: Supplementary file 2 — Additional file 1: Table S1. Analysis of association between TRIM15 expression and clinicopathological parameters in NSCLC. [file 12964_2022_875_MOESM2_ESM.docx]

**Additional file 1: Table S1. Analysis of association between TRIM15 expression and clinicopathological parameters in lung cancer**

|  |  | TRIM15 expression | |  |
| --- | --- | --- | --- | --- |
| Characteristics | **N** | **Low** | **High** | ***P* value** |
| Age |  |  |  | 0.667 |
| ≤ 60 | 40 | 19 | 21 |  |
| > 60 | 58 | 25 | 33 |  |
| Gender |  |  |  | 0.059 |
| Male | 41 | 23 | 18 |  |
| Female | 57 | 21 | 36 |  |
| Differentiation |  |  |  | 0.560 |
| Well | 20 | 11 | 9 |  |
| Moderate | 56 | 23 | 33 |  |
| Poor | 22 | 10 | 12 |  |
| Tumor size (T) |  |  |  | 0.004 |
| T1-T2 | 36 | 23 | 13 |  |
| T3-T4 | 62 | 21 | 41 |  |
| Lymph node metastasis |  |  |  | 0.002 |
| N0-N1 | 41 | 26 | 15 |  |
| N2-N3 | 57 | 18 | 39 |  |
| Distant metastasis (M) |  |  |  | 0.003 |
| Negative (M0) | 81 | 42 | 39 |  |
| Positive (M1) | 17 | 2 | 15 |  |
| Tumor stage |  |  |  | 0.010 |
| Ⅰ-Ⅱ | 46 | 27 | 19 |  |
| Ⅲ-Ⅳ | 52 | 17 | 35 |  |

**P* values <0.05 were considered statistically significant (chi-square test for categorical variables).
